# A modifiable imaging biomarker: epicardial adipose tissue density in ischemia with non-obstructive coronary arteries

**DOI:** 10.3389/fcvm.2026.1686602

**Published:** 2026-01-30

**Authors:** Fan Sun, Yu Tian, Wenji Yu, XuHong Song, Feifei Zhang, Jianfeng Wang, Xiaoliang Shao, Bao Liu, Xiaoyu Yang, Peng Wan, Yongjun Chen, Sijin Li, Yuetao Wang

**Affiliations:** 1 Changzhou Medical Center, Nanjing Medical University, Changzhou, Jiangsu, China; 2 The First People’s Hospital of Changzhou, Changzhou, Jiangsu, China; 3 Department of Nuclear Medicine, The Third Affiliated Hospital of Soochow University, Changzhou, Jiangsu, China; 4 Department of Cardiology, The Third Affiliated Hospital of Soochow University, Changzhou, Jiangsu, China; 5 Department of Nuclear Medicine, First Hospital of Shanxi Medical University, Taiyuan, Shanxi, China

**Keywords:** coronary artery disease, epicardial adipose tissue, ischemia with non-obstructive coronary arteries, myocardial ischemia, statin

## Abstract

**Background:**

The impact of epicardial adipose tissue (EAT) on the risk of non-obstructive coronary artery disease (CAD) remains unclear. This study aims to investigate the association between EAT and ischemia with non-obstructive coronary arteries (INOCA).

**Methods:**

This study enrolled 281 patients with angina or other symptoms suggestive of myocardial ischemia who underwent single-photon emission computed tomography myocardial perfusion imaging (SPECT-MPI). All patients had confirmed non-obstructive coronary artery disease (stenosis <50%) by either coronary angiography (CAG) or coronary CT angiography (CCTA) within 3 months before or after MPI. Based on MPI results, patients were categorized into ischemic and non-ischemic groups. Epicardial adipose tissue (EAT) density and volume were measured, and relevant clinical parameters were collected for analysis.

**Results:**

The results revealed that 37.72% of the patients had INOCA, and these patients exhibited significantly higher body mass index (BMI) and EAT density. No statistically significant difference in EAT volume was observed between groups. Both EAT density (OR = −1.846, 95% CI: 1.353–2.559, *p* < 0.05) and volume (OR = −1.703, 95% CI: 1.151–2.551, *p* < 0.05) were identified as independent risk factors for INOCA. Furthermore, EAT density demonstrated a linear relationship with disease risk. In statin users, the positive association between EAT density and INOCA was attenuated. (*β* = −0.039, *p* = 0.046).

**Conclusions:**

EAT density is an independent risk factor for INOCA, with its increase showing a linear association with INOCA risk. Further, statin use was associated with a reduction in this EAT density-related INOCA risk.

## 1 Introduction

INOCA (Ischemia with No Obstructive Coronary Arteries) is defined as a condition characterized by ischemic chest pain and objective evidence of myocardial ischemia, but without obstructive coronary artery stenosis (≥50% stenosis). Globally, approximately 112 million patients suffer from angina, and up to 70% of those undergoing invasive angiography show no evidence of obstructive coronary artery disease, with a significant proportion of symptoms attributed to INOCA (1, 2). Compared to patients with no ischemia, those with ischemia exhibit a poorer prognosis and a higher risk of adverse cardiovascular events (3). According to the 2019 ESC Guidelines for the Diagnosis and Management of Chronic Coronary Syndromes, first-line evaluation of angina should prioritize non-invasive testing (4). Single-photon emission computed tomography myocardial perfusion imaging (SPECT-MPI), as a highly accurate and evidence-based non-invasive technique, has been widely used for the diagnosis of myocardial ischemia and aids in the diagnosis of INOCA (5). However, our understanding of INOCA remains limited, and there is an urgent need to explore modifiable risk factors to enable early identification, precise diagnosis, and effective treatment.

Epicardial adipose tissue (EAT) is a metabolically active visceral fat located between the myocardium and the visceral layer of the pericardium. It exerts its biological functions through the secretion of various bioactive molecules. These paracrine and endocrine factors have been demonstrated to exert pro-inflammatory effects on the vascular system, thereby contributing to the development and progression of coronary atherosclerosis and cardiovascular diseases (6). As a promising imaging biomarker, EAT is closely associated with coronary artery disease (CAD), and plays a significant role in the prediction and prognosis of CAD (7).

Studies indicate that lifestyle modifications and pharmacological therapies [e.g., statins, glucagon-like peptide-1 receptor agonists (GLP-1), and sodium-glucose cotransporter-2inhibitors (SGLT2)] significantly reduce EAT accumulation and improve cardiovascular outcomes (8, 9). Notably, statin therapy exhibits pleiotropic effects independent of lipid-lowering, including potent anti-inflammatory properties, and can reduce the fat attenuation value (10, 11), which may represent a key pathway for EAT reduction and prognostic benefit. However, the majority of studies have focused on the relationship between EAT and obstructive coronary artery disease, while significant gaps remain in understanding the association between EAT and INOCA.

EAT can be quantitatively characterized by tissue density and volume, which are typically assessed using computed tomography (CT) imaging (12). Specifically, current research has primarily focused on EAT volume, which demonstrates significant associations with higher Coronary Artery Calcium scores (CACS), coronary artery stenosis, obstructive CAD and major adverse cardiac events (MACE) (13, 14). EAT volume reflects the long-term cumulative pathological effects, and its association with non-obstructive CAD remains controversial. Current studies have demonstrated that EAT density is also associated with cardiovascular risk factors (15). Furthermore, EAT density captures the comprehensive characteristics of EAT (6), reflecting both the functional status and inflammatory level of adipose tissue (16, 17). This enables a more dynamic assessment of disease activity, with changes possibly occurring earlier than alterations in volume or the emergence of clinical symptoms, thereby more dynamically reflecting disease activity. The process is consistent with the pathogenesis of INOCA, which suggests that EAT density may have a closer relationship with it. Therefore, this study mainly investigates the relationship between EAT density and INOCA.

## 2 Methods

### 2.1 Study population

The present study enrolled 281 patients who underwent single-photon emission computed tomography myocardial perfusion imaging (SPECT-MPI) at Changzhou First People's Hospital between January 1, 2018 and December 31, 2024 for suspected myocardial ischemia symptoms such as angina pectoris. The study included patients who had undergone either coronary angiography (CAG) or coronary computed tomography angiography (CCTA) within three months before or after the MPI examination, showing non-obstructive coronary artery disease (stenosis < 50%). Traditional cardiovascular risk factors were systematically collected through the hospital's electronic medical record system, including hypertension, diabetes mellitus (DM), dyslipidemia, body mass index (BMI), and active smoking. Hypertension was defined by either sustained elevated blood pressure (systolic pressure ≥140 mmHg and/or diastolic pressure ≥90 mmHg) or current use of antihypertensive pharmacotherapy. DM was diagnosed according to either elevated random plasma glucose levels (≥8.0 mmol/L) or previously established clinical diagnosis. Hyperlipidemia was identified based on documented medical history, abnormal lipid profile (total cholesterol concentration >5.2 mmol/L or low-density lipoprotein cholesterol level >3.36 mmol/L), or ongoing lipid-lowering treatment. Active smoking was defined as smoking within past 6 months. Height and weight were measured to ascertain BMI. Blood test indicators collected within one week of examination included, total cholesterol (TC), triglycerides (TG), high-density lipoprotein cholesterol (HDL-C), low-density lipoprotein cholesterol (LDL-C), Creatinine, B-type Natriuretic Peptide (BNP), Cardiac Troponin I (cTnI), C-reactive protein (CRP), White Blood Cell Count (WBC), Neutrophils. Statin use was defined as regular statin therapy for at least 30 days prior to the MPI examination.

**Figure 1:**
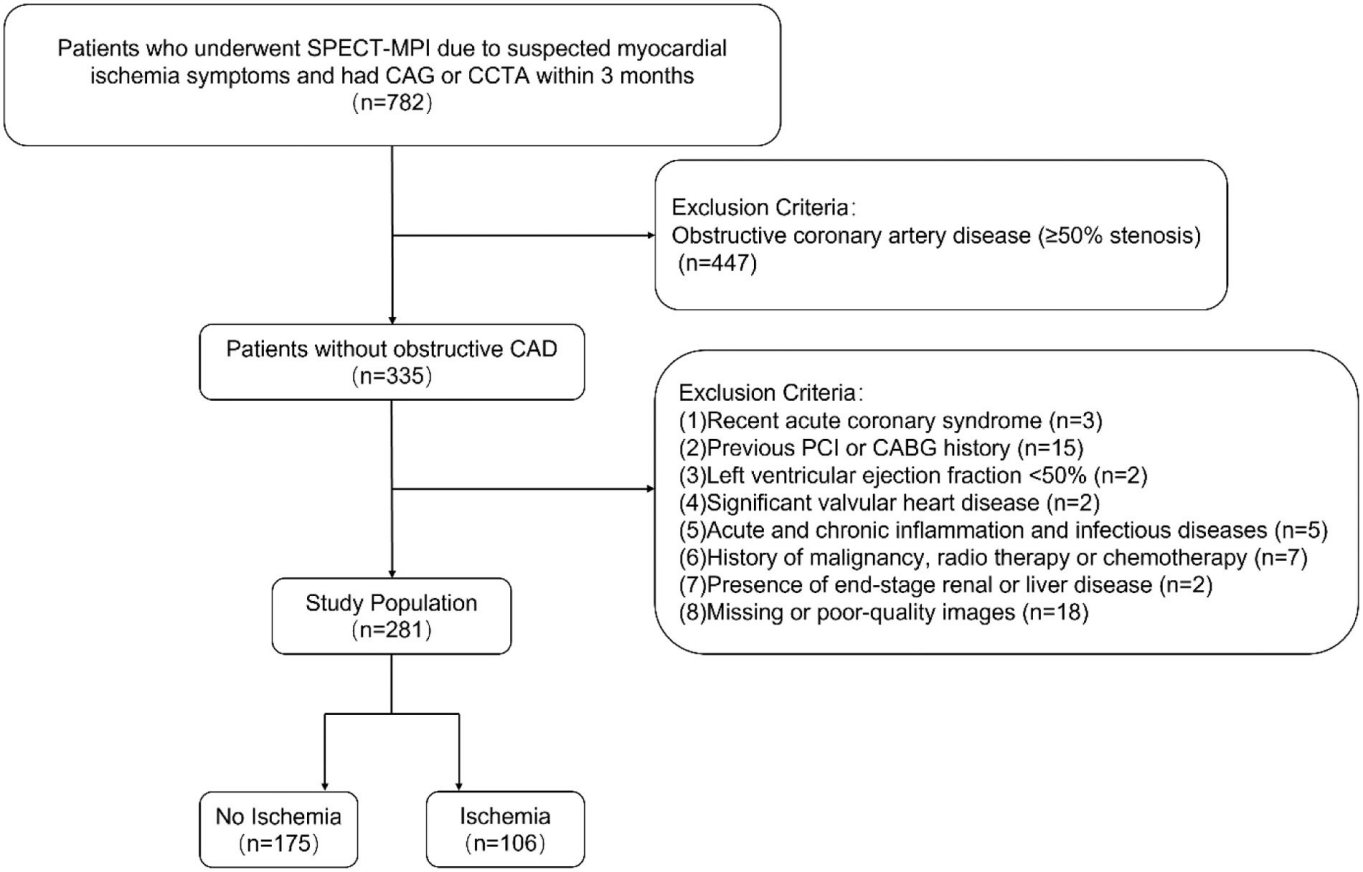
Flowchart of population selection. SPECT-MPI, single-photon emission computed tomography myocardial perfusion imaging; CAG, coronary angiography; CCTA, coronary computed tomography angiography; CAD, coronary artery disease; PCI, percutaneous coronary intervention; CABG, coronary artery bypass grafting.

The exclusion criteria were as follows: (1) Obstructive coronary artery disease (≥50% stenosis); (2) Recent acute coronary syndrome (within 3 months); (3) History of previous percutaneous coronary intervention or coronary artery bypass grafting; (4) Left ventricular ejection fraction <50%; (5) Significant valvular heart disease requiring surgical intervention; (6) Acute and chronic inflammation and infectious diseases; (7) History of malignancy, radiotherapy or chemotherapy; (8) Presence of end-stage renal or liver disease, life expectancy <4 years; (9) Missing or poor-quality images. Figure 1 illustrates the study population selection flowchart. The study protocol was approved by the Medical Ethics Committee of The First People's Hospital of Changzhou (Approval No. 2025026). Informed consent was waived for the overall study due to its retrospective and observational nature, with all data being anonymized. However, separate explicit consent was obtained for the use of any identifiable typical case materials.

The Pre-test Probability (PTP) (18) was calculated using the CAD Consortium clinical score. The model input variables included age, sex, chest pain type, smoking history, diabetes, dyslipidemia, and family history of CAD. The final PTP was stratified into low (<15%), intermediate (15%–65%), and high (>65%) risk groups. The mean PTP was 0.3156 ± 0.2224, with 71.5% of patients classified as intermediate or high risk.

### 2.2 Image acquisition and diagnosis of myocardial ischemia

The examination was performed using a Symbia T16 dual-detector SPECT/CT system (Siemens, Germany). Patients were instructed to refrain from using nitrates and β-blockers for 24 h prior to the test. Each patient underwent a two-day stress-rest myocardial perfusion imaging (MPI) using 99mTc-MIBI (provided by Jiangsu Xinke Pharmaceutical Co., Ltd.) as the imaging agent, with a radiochemical purity of >95%. The injection dose ranged from 740 to 925 MBq, and MPI was performed 60–90 min after injection (19). The primary stress method was the exercise stress test, performed in 231 cases (82.2%). For patients unsuitable for exercise, adenosine pharmacological stress testing was used, totaling 50 cases (17.8%). The image acquisition parameters were as follows: magnification factor 1.45, matrix size 128 × 128, energy peak 140 keV, and a 20% window width. The projection data were filtered using the 3D Flash iterative method (with 16 iterations and 2 subsets), resulting in the three-dimensional axial images of the left ventricle (short axis, horizontal long axis, and vertical long axis).

Visual assessment was used to evaluate the perfusion score of each myocardial segment in the reconstructed MPI. Myocardial perfusion images were reconstructed and analyzed using dedicated software, dividing the left ventricular myocardium into 17 segments, and were assessed using a 5-point scoring system across 17 segments (0 = normal perfusion; 1 = mild perfusion abnormality; 2 = moderate perfusion abnormality; 3 = severe perfusion abnormality; 4 = no perfusion). The scores of myocardial segments with abnormalities during stress and rest MPI were summed to obtain the summed stress score (SSS) and summed rest score (SRS), respectively. The summed difference score (SDS) was calculated by subtracting SRS from SSS. Myocardial ischemia was defined as the presence of reversible perfusion defects, specifically an SDS ≥ 2 (20). This process was performed jointly by two experienced nuclear medicine physicians. In cases of disagreement, a third senior physician was consulted, and a consensus diagnosis was reached through discussion.

### 2.3 Measurements of EAT density, volume and CACS

Following the acquisition of stress MPI, a low-dose non-contrast chest CT scan was conducted for obtaining EAT density, EAT volume and CACS. Non-contrast CT scans were performed using retrospective ECG-gating, with an integrated SPECT/CT system following the acquisition of gated myocardial perfusion imaging (MPI) images. The CT scanning parameters were as follows: phase window: 60%–80% of the RR interval, tube voltage: 130 kV, tube current: 100 mAs, and slice thickness: 3 mm. The scan coverage extended from the level of the tracheal carina to approximately 1–2 cm below the diaphragmatic surface of the heart, covering a total length of about 20 cm. EAT was defined as the adipose tissue located between the myocardium and the pericardium, with a window width of −190 to −30 Hounsfield Units (HU). Using reconstructed axial scans, two independent observers manually delineated the EAT from the level of the pulmonary artery bifurcation to the diaphragm at 5-mm intervals. The EAT volume (calculated as the sum of the cross-sectional area of fat multiplied by the CT slice thickness) and the EAT density were automatically obtained using software (Syngo Volume; Siemens Medical Solutions) (21). The final EAT density and EAT volume were determined by averaging the measurements from the two observers. The intra-class correlation coefficient was 0.968 (95% CI: 0.952–0.979, *P* < 0.001). The CACS is defined as the sum of the calcium scores of each coronary artery, obtained using Agatston automated analysis software (22). Then the CACS was divided into a no calcification group (CACS = 0) and a calcification group (CACS > 0).

### 2.4 Definition of non-obstructive CAD

CCTA was performed using a Somatom Definition Flash dual-source spiral CT scanner (Siemens, Germany). 186 (66.2%) patients received CAG, and CAG was carried out through radial or femoral artery access utilizing the Seldinger technique and the Judkins method. Two experienced cardiologists independently assessed the degree of stenosis in the left main (LM), left anterior descending (LAD), left circumflex (LCX), and right coronary arteries (RCA). A third cardiologist was consulted to reach a consensus when discrepancies arose. In this study, non-obstructive coronary artery disease (Non-Obstructive CAD) was defined as the presence of <50% stenosis (including 0% stenosis) in all four coronary arteries (LM, LAD, LCX, and RCA) (23, 24).

### 2.5 Definition of INOCA

The diagnostic criteria for INOCA in this study are as follows: (1) Presence of ischemic chest pain symptoms; (2) Objective evidence of myocardial ischemia (reversible perfusion defects on MPI); (3) CAG or CCTA showing coronary artery stenosis of less than 50% (24).

### 2.6 Radiation exposure

The dose for a non-contrast CT scan to assess CACS, EAT density and EAT volume was 1–2 mSv.

### 2.7 Statistical analysis

Data analysis was performed using The R Programming Language (version 4.4.2) and IBM SPSS Statistics (version 27.0.1). For variables with missing data rates <20%, multiple imputation was conducted using R software. We compared the baseline characteristics of patients with and without ischemia based on MPI diagnosis. Continuous variables are presented as mean ± SD when normally distributed and as median (25th–75th percentiles) when not normally distributed. Normality of each variable was assessed using the Kolmogorov–Smirnov test. The Mann–Whitney U test, independent-samples t-test, and *χ*² test were performed as appropriate. Categorical variables are expressed as numbers (%), and analysis was conducted using the *χ*² test or Fisher's exact test, as appropriate. Previously reported risk factors for myocardial ischemia were retained as confounding factors and included in both univariable and multivariable regression analyses. Collinearity among significant independent variables was assessed, and only variables with a variance inflation factor (VIF) of less than 5 were retained. The relationship between EAT density and INOCA was assessed using Generalized Additive Models (GAM) and smooth curve fitting. Spearman correlations evaluated relationships between EAT density and Age, Men, BMI, Active Smoking, Hypertension, DM, Hyperlipidemia, TC, TG, LDL-C, HDL-C, BNP, cTnI, CRP, EAT volume. The moderating effect was assessed using regression analysis.

## 3 Results

### 3.1 Patient characteristics

The baseline characteristics of all patients are presented in Table 1. Overall, the mean age was 59.51 ± 10.63 years, 142 (50.5%) were male, and the mean BMI was 24.82 ± 3.22 kg/m². The mean EAT density was −77.47 ± 3.64 HU (range: −66.7 to −86.8 HU). Among them, 106 patients (37.7%) had ischemia, i.e., INOCA. Compared to patients with no ischemia, INOCA patients had higher BMI (25.35 ± 3.58 kg/m² vs. 24.50 ± 2.95 kg/m², *P* < 0.05) and EAT density (−76.69 ± 3.77 HU vs. −77.47 ± 3.64 HU, *P* < 0.05). In addition, BNP [70.00 (47.00–177.00) ng/L vs. 70.00 (33.48–117.25) ng/L, *P* < 0.05], cTnI [0.0037 (0.0023–0.0065) mg/L vs. 0.0029 (0.0020–0.0045) mg/L, *P* < 0.05] are slightly elevated, while CRP [1.07(0.50–2.70) mg/L vs. 2.80 (0.50–4.28) mg/L, *P* < 0.05] is slightly reduced, but all are within the normal range. No significant difference was observed in EAT volume and statin usage.

**Table 1:** Baseline patient characteristics.

Characteristics	Total (*n* = 281)	No ischemia (*n* = 175)	Ischemia (*n* = 106)	*P* value
Age, y	60.00 (53.00–68.00)	60.00 (53.00–68.00)	59.50 (50.25–68.00)	0.712
Men, *n* (%)	142 (50.5%)	86 (49.1%)	56 (52.8%)	0.549
BMI, kg/m^2^	24.64 (22.86–26.56)	24.22 (22.66–26.03)	25.15 (23.22–27.32)	0.021*
Active Smoking, *n* (%)	97 (34.5%)	62 (35.4%)	35 (33.0%)	0.680
Hypertension, *n* (%)	167 (59.4%)	100 (57.1%)	67 (63.2%)	0.316
DM, *n* (%)	56 (19.9%)	34 (19.4%)	22 (20.8%)	0.787
Hyperlipidemia, *n* (%)	118 (42.1%)	74 (42.3%)	44 (41.9%)	0.950
TC, mmol/L	4.15 (3.53–4.85)	4.13 (3.59–4.78)	4.21 (3.44–5.00)	0.846
TG, mmol/L	1.52 (1.09–2.17)	1.56 (1.14–2.13)	1.40 (1.07–2.19)	0.504
LDL-C, mmol/L	2.29 (1.82–2.89)	2.33 (1.84–2.80)	2.24 (1.72–2.98)	0.790
HDL-C, mmol/L	1.15 (0.98–1.34)	1.13 (0.98–1.29)	1.17 (0.99–1.39)	0.261
BNP, ng/L	70.00 (40.00–129.00)	70.00 (33.48–117.25)	70.00 (47.00–177.00)	0.047*
cTnI, ng/mL	0.0031 (0.0021–0.0050)	0.0029 (0.0020–0.0045)	0.0037 (0.0023–0.0065)	0.013*
CRP, mg/L	2.10 (0.50–3.80)	2.80 (0.50–4.28)	1.07 (0.50 -2.70)	0.009*
WBC, ×10^9^/L	5.92 (5.01–6.82)	5.98 (5.17–6.95)	5.56 (4.90–6.65)	0.063
Neutrophils, ×10^9^/L	3.71 (2.99–4.44)	3.78 (3.11–4.46)	3.60 (2.80–4.37)	0.137
Calcification, *n* (%)	75 (26.7%)	47 (26.9%)	28 (26.4%)	0.935
EAT volume, cm^3^	138.61 ± 46.92	135.38 ± 44.90	143.94 ± 49.84	0.139
EAT density, HU	−77.47 ± 3.64	−77.94 ± 3.49	−76.69 ± 3.77	0.005*
Satin, *n* (%)	71 (25.3%)	41 (23.43%)	30 (28.30%)	0.362

BMI, body mass index; DM, diabetes mellitus; TC, total cholesterol; TG, triglyceride; LDL-C, low-density lipoprotein cholesterol; HDL-C, high-density lipoprotein cholesterol; BNP, B-type Natriuretic Peptide; cTnI, Cardiac Troponin I; CRP, C-reactive protein; WBC, White Blood Cell Count; EAT, epicardial adipose tissue.

* *P* < 0.05.

### 3.2 Baseline characteristics stratified by tertiles of EAT density

Participants were stratified into tertiles based on EAT density (low, medium, and high), and their baseline characteristics were compared (Table 2). The mean EAT densities in the low-, medium-, and high-density groups were −81.50 ± 1.70 HU, −77.46 ± 0.92 HU, and −73.48 ± 1.89 HU, respectively. Compared to the other groups, patients with high EAT density were younger (58.00 y), had lower TG levels (1.32 mmol/L), cTnI levels (0.0027 ng/mL), and EAT volume (111.89 cm^3^), but a significantly higher incidence of myocardial ischemia (47.87%), and all *p* < 0.05.

**Table 2:** Baseline characteristics stratified by tertiles of EAT density.

Characteristics	Bottom tertile (*n* = 93)	Middle tertile (*n* = 94)	Top tertile (*n* = 94)	*P* value for trend
EAT density, HU	−81.50 ± 1.76	−77.46 ± 0.92	−73.48 ± 1.89	<0.001*
Age, y	62.00 (57.00–69.00)	58.50 (53.00–67.00)	58.00 (48.50–67.00)	0.015*
Men, *n* (%)	45 (48.39%)	47 (50.00%)	47 (50.00%)	0.968
BMI, kg/m^2^	25.04 (23.32–26.67)	24.10 (22.06–25.95)	24.66 (22.88–26.83)	0.043
Active Smoking, *n* (%)	33 (35.48%)	33 (35.11%)	31 (32.98%)	0.927
Hypertension, *n* (%)	60 (64.52%)	51 (54.26%)	56 (59.57%)	0.360
DM, *n* (%)	18 (19.35%)	19 (20.21%)	19 (20.21%)	0.986
Hyperlipidemia, *n* (%)	41 (44.09%)	40 (42.55%)	38 (40.43%)	0.879
TC, mmol/L	3.98 (3.45–4.69)	4.21 (3.65–4.93)	4.28 (3.54–4.76)	0.427
TG, mmol/L	1.62 (1.24–2.45)	1.58 (1.25–2.20)	1.32 (0.94–1.82)	<0.001*
LDL-C, mmol/L	2.20 (1.72–2.76)	2.38 (1.85–3.02)	2.26 (1.84–2.88)	0.243
HDL-C, mmol/L	1.12 (0.98–1.29)	1.15 (0.97–1.31)	1.16 (1.02–1.37)	0.200
BNP, ng/L	70.00 (32.42–122.75)	70.00 (35.88–95.50)	83.00 (56.50–170.00)	0.065
cTnI, ng/mL	0.0036 (0.0023–0.0058)	0.0029 (0.0019–0.0051)	0.0027 (0.0020–0.0044)	0.042*
CRP, mg/L	1.90 (0.50–3.20)	2.60 (0.50–4.60)	1.84 (0.50–3.38)	0.519
EAT volume, cm^3^	160.43 (134.09–200.44)	128.91 (100.68–150.87)	111.89 (86.35–142.66)	<0.001*
Ischemia, *n* (%)	30 (32.26%)	31 (32.98%)	45 (47.87%)	0.045*

BMI, body mass index; DM, diabetes mellitus; TC, total cholesterol; TG, triglyceride; LDL-C, low-density lipoprotein cholesterol; HDL-C, high-density lipoprotein cholesterol; BNP, B-type Natriuretic Peptide; cTnI, Cardiac Troponin I; CRP, C-reactive protein; WBC, White Blood Cell Count; EAT, epicardial adipose tissue.

* *P* < 0.05.

### 3.3 Univariable and multivariable logistic regression analysis between EAT density and INOCA

Univariable logistic regression analysis (Table 3) revealed that a per-SD increase in EAT density was independently associated with INOCA (OR, 1.421; 95% CI, 1.109–1.835; *P* < 0.05), and the OR for BMI was 1.086 (95% CI: 1.006–1.172, *P* < 0.05). In the multivariable logistic regression analysis adjusted for age, sex, BMI, active smoking, hypertension, diabetes mellitus, hyperlipidemia, and EAT volume, both EAT metrics remained independently associated with INOCA. For EAT density, a per-SD increase (1SD = 3.64 HU) was associated with 84.6% higher odds of INOCA (OR = 1.846, 95% CI: 1.013–2.559, *P* < 0.01). Similarly, a per-SD increase (1SD = 46.92 cm^3^) in EAT volume showed a 70.3% increase in the odds of INOCA (OR = 1.703, 95% CI: 1.151–2.551, *P* < 0.001).

**Table 3:** Univariable and multivariable logistic regression analysis between EAT density and INOCA.

Variable	Univariable analysis		Multivariable analysis	
	OR (95%CI)	*P* value	OR (95%CI)	*P* value
Age	0.993 (0.970, 1.015)	0.521		
Men	0.863 (0.532, 1.398)	0.549		
BMI	1.086 (1.006, 1.172)	0.034*		
Active Smoking	1.113 (0.669, 1.853)	0.681		
Hypertension	0.776 (0.473, 1.274)	0.316		
DM	0.921 (0.505, 1.678)	0.787		
Hyperlipidemia	1.016 (0.622, 1.658)	0.950		
EAT volume (per SD)	1.200 (0.942, 1.531)	0.139	1.703 (1.1502.551)	0.009*
EAT density (per SD)	1.421 (1.109, 1.835)	0.006*	1.846 (1.353, 2.559)	<0.001*

BMI, body mass, index; DM, diabetes mellitus; EAT, epicardial adipose tissue; OR, odds ratio.

* *P* < 0.05.

### 3.4 The relationship between EAT density and INOCA

**Figure 2:**
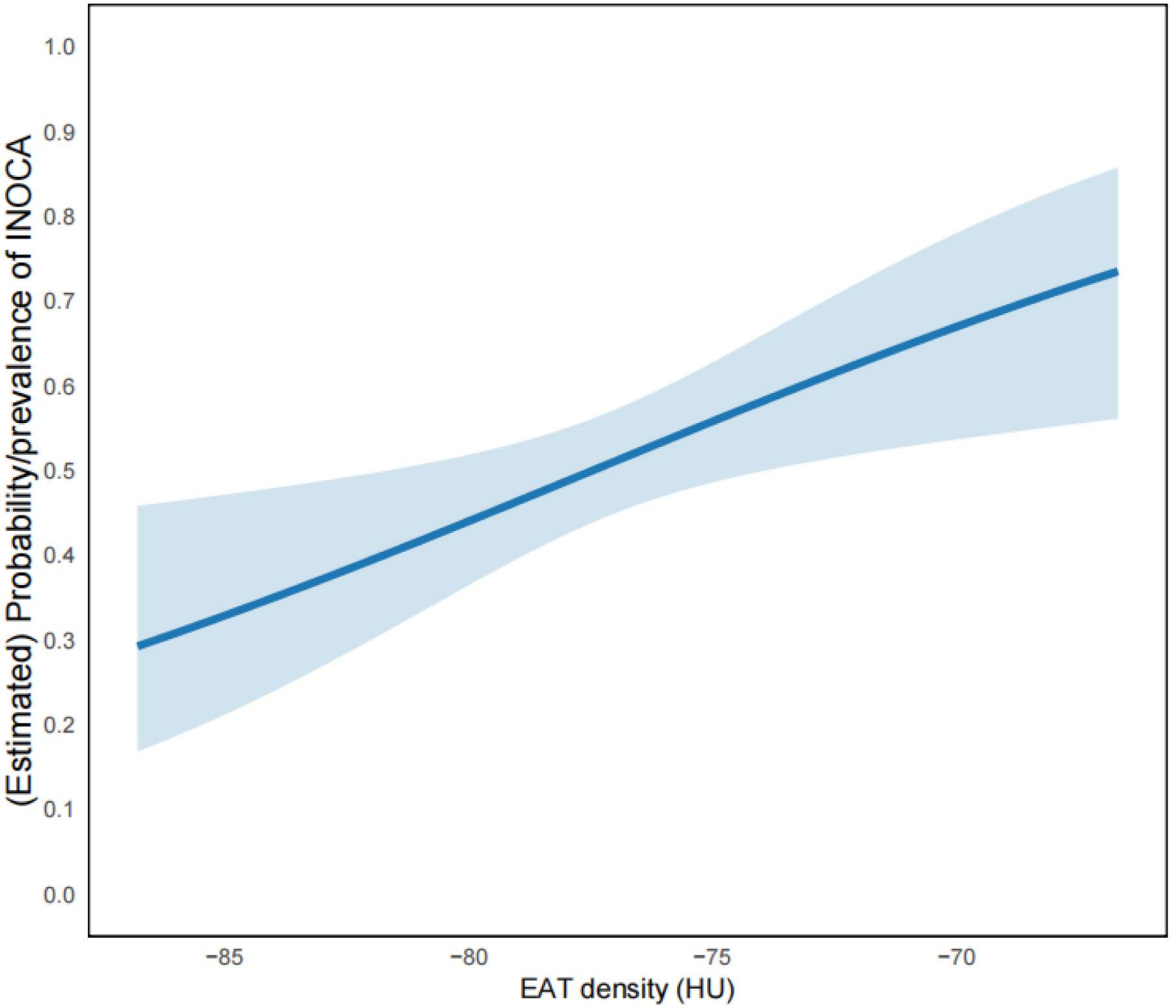
Generalized additive models to validate the relationship between EAT density and INOCA. The *Y*-axis represents the prevalence of INOCA occurrence, and the *X*-axis represents EAT density (in Hounsfield Units, HU). The results show that after adjusting for factors such as age, sex, BMI, hypertension, diabetes, hyperlipidemia, and EAT volume, the relative risk of INOCA gradually increases with higher EAT density.

After adjusting for factors such as age, sex, BMI, hypertension, DM, hyperlipidemia, and EAT volume, we used a Generalized Additive Model (GAM) to assess the correlation between EAT density and INOCA (Figure 2). The smooth term was close to linear (edf = 1.001, *p* = 0.004), and the nonlinearity test was nonsignificant (*p* = 0.254), indicating a linear relationship between EAT density and INOCA.

### 3.5 Correlation analysis of EAT density with cardiology risk markers

Table 4 indicates that EAT density was inversely correlated with age, TG and EAT volume, and positively correlated with HDL-C and cTnI. The correlation coefficients ranged from 0.143 to 0.496 (*P* < 0.05), indicating weak-to-moderate associations between these parameters and EAT density.

**Table 4:** Correlation analysis of EAT density with cardiology risk markers.

Variable	*R* value	*P* value
Age	−0.172	0.004*
Men	.017	0.780
BMI	−0.055	0.361
Active smoking	−.013	0.822
Hypertension	−.040	0.506
DM	−.028	0.639
Hyperlipidemia	−.043	0.469
TC	0.023	0.706
TG	−0.197	<0.001*
LDL-C	0.047	0.437
HDL-C	0.132	0.027*
Creatinine	−0.069	0.252
BNP	0.138	0.064
cTnI	0.143	0.017*
CRP	−0.019	0.824
EAT volume	−0.496	<0.001*

BMI, body mass index; DM, diabetes mellitus; TC, total cholesterol; TG, triglyceride; LDL-C, low-density lipoprotein cholesterol; HDL-C, high-density lipoprotein cholesterol; BNP, B-type Natriuretic Peptide; cTnI, Cardiac Troponin I; CRP, C-reactive protein; EAT, epicardial adipose tissue.

* *P* < 0.05.

### 3.6 Differences in EAT density between statin users and non-users

**Figure 3:**
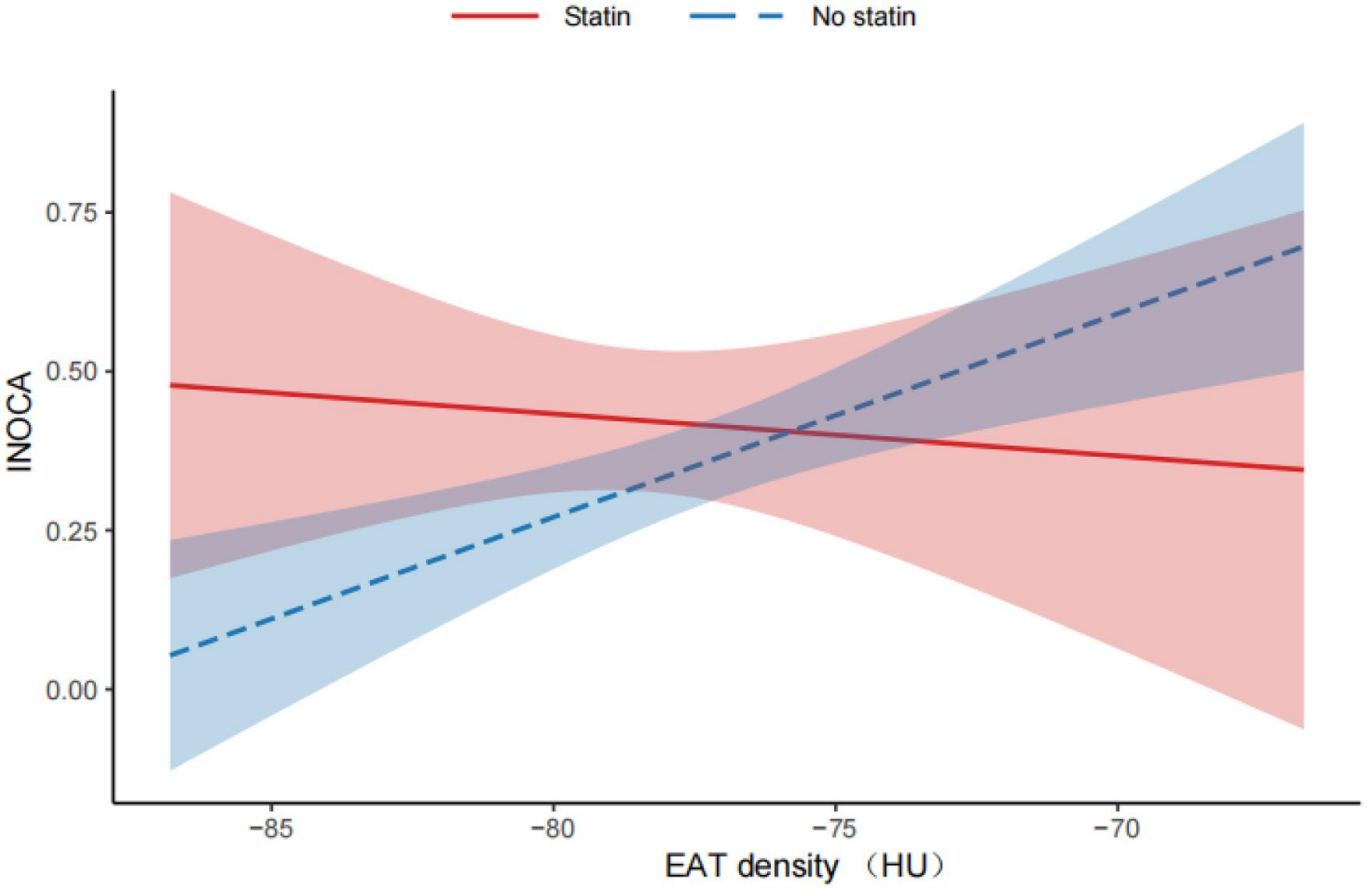
Moderating effect of EAT density on INOCA.

In this study, Statin-treated patients showed significantly lower EAT density values (−78.39 ± 3.34 HU vs. −77.15 ± 3.69 HU, *P* = 0.013). The moderation analysis demonstrated a statistically significant moderating effect of statin use on the association between EAT density and INOCA risk (interaction term *β* = −0.039, *p* = 0.046). Simple slope analysis (Figure 3) further revealed that EAT density was significantly positively associated with INOCA risk in non-statin users (*β* = 0.032, *p* < 0.001), while this association was markedly attenuated in statin-treated patients (*β* = −0.007, *p* = 0.70).

**Figure 4:**
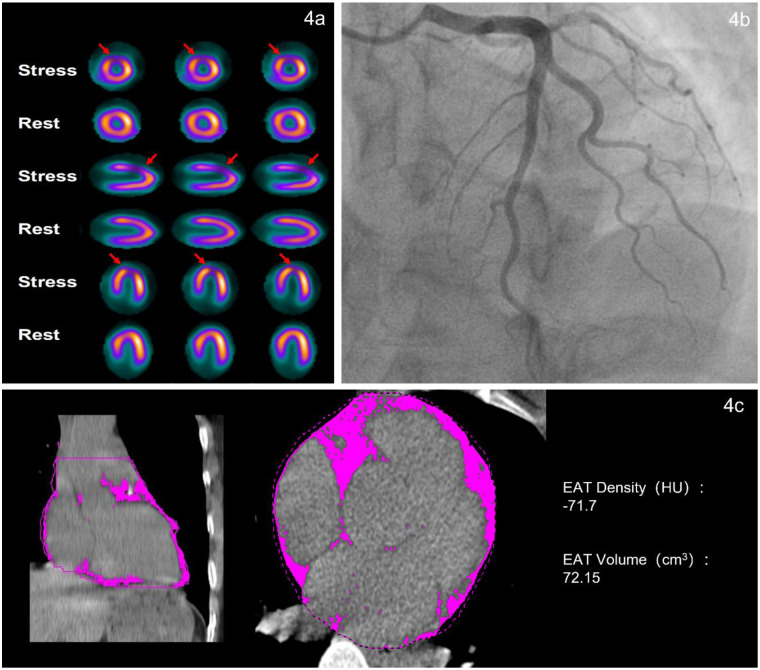
Typical case of patient with INOCA. The patient is a 43-year-old female admitted to the hospital due to chest tightness for 2 months. The EAT density is −71.7 HU. Blood test indicators showed CRP 0.98 mg/L, BNP 92 ng/L, and cTnI0.0021 ng/mL. **(a)** Stress-rest myocardial perfusion imaging revealed reversible myocardial ischemia in the partial anterior wall, apex, and anterior septal wall of the left ventricle. **(b)** CAG revealed no significant stenosis (<50% luminal narrowing) in LAD, LCX or RCA. **(c)** Non-contrast CT scan shows cross-sectional and coronal views of epicardial adipose tissue (the pink area).

**Figure 5:**
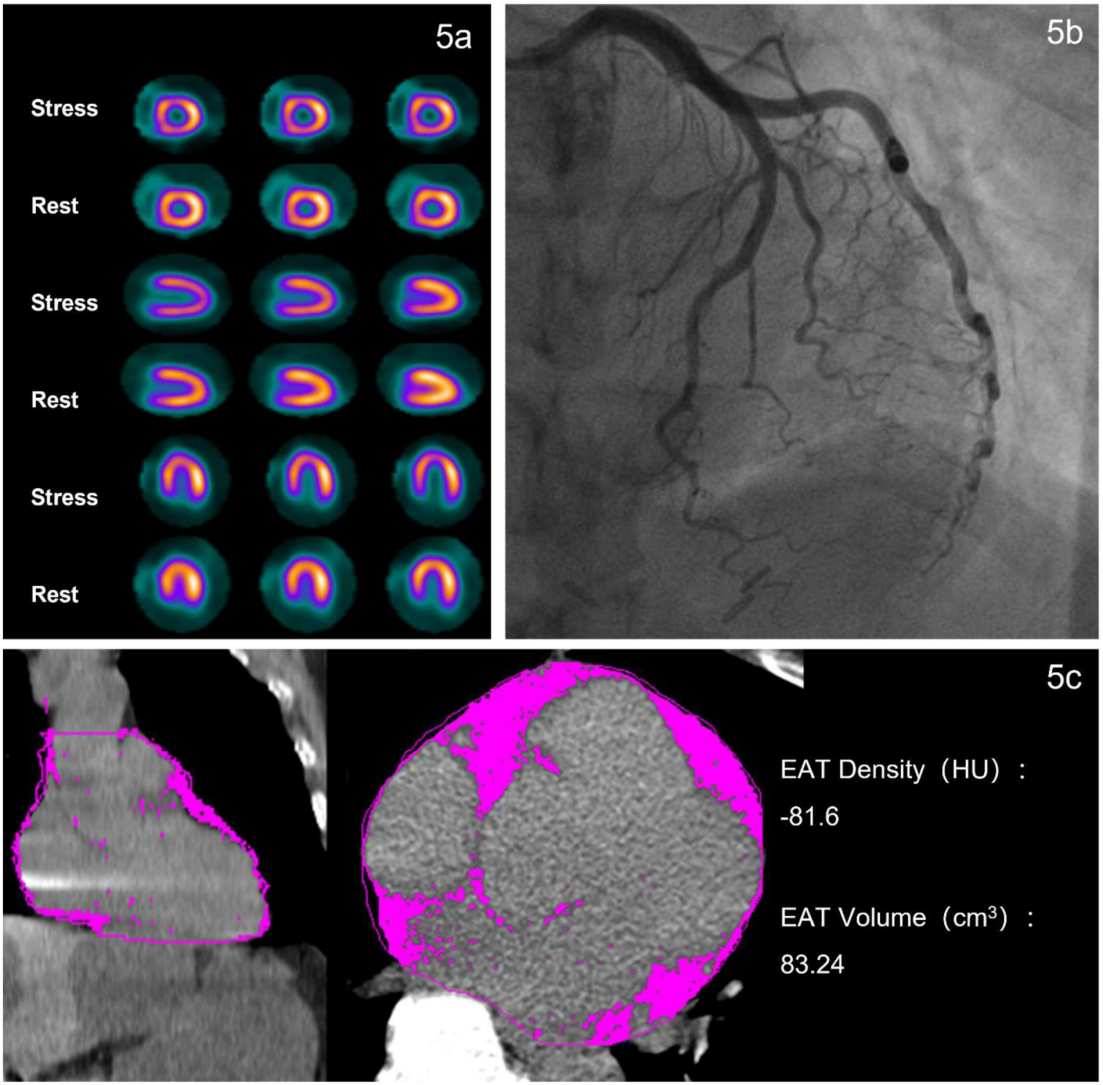
Typical case of patient with no ischemia. A 57-year-old female patient was admitted to the hospital due to chest pain for 2 months. Blood test indicators showed CRP 0.5 mg/L, BNP 31.3 ng/L, and cTnI0.0035 ng/mL. **(a)** The examination results showed no significant myocardial ischemia on stress-rest myocardial perfusion imaging. **(b)** CAG revealed 10% stenosis in the proximal segment of RCA, with no significant stenosis in LAD or RCA. **(c)** Non-contrast CT scan displayed the coronal and axial views of EAT (the pink area), with EAT density of −81.6 HU.

## 4 Discussion

Our research findings indicate that: (1) Compared to patients without ischemia, patients with ischemia have higher EAT density. (2) EAT density represents an independent risk factor for INOCA, distinct from traditional cardiovascular risk factors and EAT volume, and shows a linear association with INOCA risk. (3) Statin-treated patients exhibited lower EAT density, and in these patients, the association between EAT density and INOCA risk appeared attenuated.

INOCA patients exhibit a high clinical risk, characterized by elevated morbidity and an increased risk of major adverse cardiac events (25), and coronary microvascular dysfunction (CMD) should be recognized as the main cause of INOCA (26). While BNP, cTnI, and CRP are associated with cardiac remodeling, coronary artery disease risk, and the degree of systemic inflammation (27–29), they lack specificity and cannot fully reflect the coronary microvasculature activity, making it difficult to use them as standalone diagnostic criteria (30, 31). This study also found that although BNP, cTnI, and CRP levels showed statistically significant differences between the INOCA and non-INOCA groups, all biomarker measurements remained within their respective normal reference ranges. This indicates that conventional biomarkers have limited utility in elucidating the pathophysiological mechanisms of INOCA. Therefore, it is essential to explore novel biomarkers or evaluation systems that can more precisely explain the underlying mechanisms of INOCA occurrence.

EAT covers approximately 80% of the heart's surface and shares a microcirculatory system with the heart. As a unique visceral fat depot, EAT can release pro-inflammatory and pro-growth cytokines (e.g., TNF-α, IL-6) through endocrine and paracrine mechanisms. These cytokines enhance fatty acid metabolism and the transcription of related genes, while also regulating the expression of genes associated with inflammation and endothelial function (6, 32). Studies have shown that excessive accumulation of EAT promotes the formation of MVO in STEMI patients, with the mechanism involving EAT-derived extracellular vesicles (EVs) that mediate macrophage polarization toward the M1 phenotype, thereby exacerbating inflammatory injury (33). This also indicates that EAT primarily influences the development of CMD through inflammatory pathways (34). On CT imaging, the attenuation range of fat typically falls between −190 and −30 Hounsfield Units (HU). A less negative value (closer to 0) indicates higher density and greater attenuation. The radiological density of fat on CT is influenced by several factors, including adipocyte hypertrophy, hyperplasia, and fibrosis. Specifically, adipocyte hypertrophy and hyperplasia typically lead to fat accumulation, characterized by lower density (more negative attenuation values) (35, 36), and the effects of inflammation and the process of fibrosis lead to an increase in tissue density (37). As shown in Figure 4 and Figure 5, the EAT volume and density were significantly different between INOCA patients and non-ischemic controls. In patients with INOCA, we have observed an increase in EAT density and the possible mechanism involved may include the presence of systemic and local inflammation (38). This inflammatory response could potentially impair the endothelial vasodilation function, thereby affecting the normal function of blood vessels. Concurrently, the release of vasoactive peptides during the inflammatory process, such as endothelin-1 (ET-1), may induce coronary artery spasm, ultimately leading to myocardial ischemia (39). Notably, age, TG, EAT volume, HDL-C and cTnI were all significantly associated with EAT density in our cohort. Other study also found EAT density was associated with HDL-C and TG (40). These findings reinforcing the potential role of EAT density as a functional biomarker mediating cardiovascular effects.

Several studies corroborating our findings suggest that elevated EAT attenuation, quantified via CT imaging, is associated with an increased risk of CAD (15, 41). Higher fat density reflects a greater proportion of brown adipose tissue (BAT), which is characterized by enhanced vascularity, reduced lipid content, and elevated mitochondrial density (42). However, under pathological conditions such as obesity or hypercholesterolemia, BAT can exhibit intrinsic pro-inflammatory activity or, via adiponectin-sensitive mechanisms—including increased hepatic lipid synthesis—it can drive pro-atherogenic processes (43). These alterations enable BAT to become part of the local microenvironment that exacerbates coronary microvascular dysfunction. Therefore, EAT density, which may serve as an imaging surrogate for these changes, is closely associated with CAD progression, severity, and adverse cardiovascular outcome risk. Targeting the inflammatory state of EAT or modulating BAT activity may represent a novel therapeutic avenue for reducing cardiovascular risk and reversing cardiac pathological changes (44).

Interestingly, some findings diverge from our results. Previous studies have demonstrated that myocardial ischemia in CAD patients was solely associated with increased EAT volume (with no significant difference in density) (45), a finding that diverges from our observations in the INOCA cohort. However, both studies consistently identified an inverse correlation between EAT density and volume. Moreover, Goeller et al. reported that lower EAT density and increased EAT volume were associated with coronary artery calcification, serum markers of plaque inflammation and MACE (46). Similarly, the study by Eisenberg et al. demonstrated that both increased volume and reduced density were independently associated with MACE (47). Therefore, the divergence between our findings and those of previous literature likely reflects fundamental differences in the stage of coronary artery disease progression among the studied populations. While the present study primarily focuses on patients with INOCA, prior investigations have largely included—or been predominantly composed of—advanced patient populations with significant obstructive lesions, calcified plaques, or myocardial infarction. Accumulating evidence suggests that coronary artery disease may progress along a continuum from non-obstructive to obstructive and from non-ischemic to ischemic conditions (48, 49). This implies that the biological properties of EAT and their association with clinical outcomes may undergo dynamic evolution across different stages of atherosclerosis. Future longitudinal studies in INOCA populations, integrating multimodal imaging and histological analysis, are needed to elucidate the pathological mechanisms underlying the dynamic changes in EAT density and to clarify its specific role within the disease continuum.

This study further elucidates the potential role of EAT in the pathogenesis and progression of INOCA. Analysis of medication status during MPI examination revealed that the statin-treated group exhibited significantly lower EAT density compared to the non-treated group. And in this study, a significant interaction was observed, wherein statin use was associated with an attenuated positive link between EAT density and INOCA risk. Furthermore, studies have shown that statins can reduce EAT density by decreasing cells, blood vessels, or inflammation, thereby reducing the metabolic activity of EAT (43). These results suggest that statins may exert beneficial effects on the coronary microvascular environment by modulating the biological properties of EAT, thereby providing additional cardiovascular protection independent of lipid-lowering. In future clinical practice, dynamically monitoring changes in EAT may contribute to more accurate risk stratification, optimization of treatment decisions, and assessment of patient prognosis.

## 5 Limitations

It is important to acknowledge the limitations of this study. First, this was a retrospective single-center study, which may have increased the risk of selection bias. Second, no follow-up was conducted, and therefore, this study cannot assess the longitudinal changes in EAT density or confirm its association with patient prognosis. Third, current research lacks quantitative coronary flow indices (such as FFR, CFR, IMR, etc.), particularly those related to microvascular dysfunction. The inclusion of these indices could more accurately clarify the etiology and intrinsic phenotypes of INOCA. Furthermore, given its strategic anatomical location and unique biological interface with the coronary vasculature, the association between peri-coronary adipose tissue (PCAT)and INOCA will be further validated in subsequent studies.

## 6 Conclusions

In summary, our findings demonstrate that EAT density is an independent determinant of INOCA, exhibiting a significant linear positive correlation with INOCA risk. Importantly, statin use is associated with a modulation of this risk association. These findings highlight the important clinical implications of EAT density: it holds promise as a novel and valuable risk-stratification tool and represents a potential therapeutic intervention target. Future research is warranted to further explore feasible strategies for targeting and modulating EAT to improve clinical outcomes in patients with INOCA.

## Data Availability

The original contributions presented in the study are included in the article/Supplementary Material, further inquiries can be directed to the corresponding author.
